# Early-Stage White Matter Lesions Detected by Multispectral MRI Segmentation Predict Progressive Cognitive Decline

**DOI:** 10.3389/fnins.2015.00455

**Published:** 2015-12-02

**Authors:** Hanna Jokinen, Nicolau Gonçalves, Ricardo Vigário, Jari Lipsanen, Franz Fazekas, Reinhold Schmidt, Frederik Barkhof, Sofia Madureira, Ana Verdelho, Domenico Inzitari, Leonardo Pantoni, Timo Erkinjuntti, the LADIS Study Group

**Affiliations:** ^1^Clinical Neurosciences, Neurology, University of Helsinki and Helsinki University HospitalHelsinki, Finland; ^2^Department of Information and Computer Science, Aalto University School of ScienceEspoo, Finland; ^3^Department of Physics, University Nova of LisbonLisbon, Portugal; ^4^Institute of Behavioural Sciences, University of HelsinkiHelsinki, Finland; ^5^Department of Neurology and MRI Institute, Medical University of GrazGraz, Austria; ^6^Department of Radiology and Neurology, VU University Medical CenterAmsterdam, Netherlands; ^7^Serviço de Neurologia, Centro de Estudos Egas Moniz, Hospital de Santa MariaLisbon, Portugal; ^8^Department of Neurological and Psychiatric Sciences, University of FlorenceFlorence, Italy

**Keywords:** executive functions, cognition, image analysis, MRI, neuropsychology, white matter lesions, small vessel disease

## Abstract

White matter lesions (WML) are the main brain imaging surrogate of cerebral small-vessel disease. A new MRI tissue segmentation method, based on a discriminative clustering approach without explicit model-based added prior, detects partial WML volumes, likely representing very early-stage changes in normal-appearing brain tissue. This study investigated how the different stages of WML, from a “pre-visible” stage to fully developed lesions, predict future cognitive decline. MRI scans of 78 subjects, aged 65–84 years, from the Leukoaraiosis and Disability (LADIS) study were analyzed using a self-supervised multispectral segmentation algorithm to identify tissue types and partial WML volumes. Each lesion voxel was classified as having a small (33%), intermediate (66%), or high (100%) proportion of lesion tissue. The subjects were evaluated with detailed clinical and neuropsychological assessments at baseline and at three annual follow-up visits. We found that voxels with small partial WML predicted lower executive function compound scores at baseline, and steeper decline of executive scores in follow-up, independently of the demographics and the conventionally estimated hyperintensity volume on fluid-attenuated inversion recovery images. The intermediate and fully developed lesions were related to impairments in multiple cognitive domains including executive functions, processing speed, memory, and global cognitive function. In conclusion, early-stage partial WML, still too faint to be clearly detectable on conventional MRI, already predict executive dysfunction and progressive cognitive decline regardless of the conventionally evaluated WML load. These findings advance early recognition of small vessel disease and incipient vascular cognitive impairment.

## Introduction

Cerebral small vessel disease (SVD) is the most common cause of vascular cognitive impairment and dementia. White matter lesions (WML) are the core marker of SVD on brain imaging, together with lacunar infarcts, microbleeds, and brain atrophy. All these findings have been shown to influence clinical and cognitive outcome (Jokinen et al., 2011, 2012; Muller et al., 2011; Poels et al., 2012). The Leukoaraiosis and Disability (LADIS) study, among other studies, has demonstrated that WML are related to cognitive decline, impaired functional abilities, depression, and gait and balance disturbances (LADIS Study Group, 2011).

Magnetic resonance imaging (MRI) has been the standard method in evaluating WML. Despite significant recent improvements in quantitative image analysis techniques, one of the major obstacles in MRI is still its finite spatial resolution, which leads to partial volume effects. Together with noise and inhomogeneity, it poses difficulties to brain segmentation techniques. Often, a careful analysis of the borders between healthy and pathological tissues is required to delineate the extent and severity of lesions, applying an implicit “decision-threshold” for lesion segmentation. Furthermore, hyperintensities in MRI seem to only represent the end-stage of the disease process. More widespread tissue damage may be associated with WML, not visible on routine MRI (Schmidt et al., 2011). There is no standard to evaluate such early stages of tissue damage, since their intensity values are not sufficiently distinct from those of normal tissues.

Most modern segmentation methods rely on prior information, such as average brain atlases (Smith et al., 2004; Ashburner and Friston, 2005; Goebel et al., 2006) or manual labeling (Wismüller et al., 2004; Lee et al., 2009; Cruz-Barbosa and Vellido, 2011). Recently, a new data-driven method for tissue segmentation has been proposed, based on a discriminative clustering (DC) strategy, in a self-supervised machine learning approach (Gonçalves et al., 2014). This method reduces the use of prior information to a minimum, and utilizes multispectral MRI data. Unlike other methods, targeting only healthy tissues (Pham and Prince, 1998; Van Leemput et al., 1999; Zhang et al., 2001; Manjón et al., 2010) or specific types of lesions (Van Leemput et al., 2001; Zijdenbos et al., 2002; Styner et al., 2008; Cruz-Barbosa and Vellido, 2011), DC allows for the study of a wide range of normal and abnormal tissue types. Another major asset of the proposed method is its ability to estimate tissue probabilities for each voxel, necessary for a suitable characterization of WML evolution. Voxels can be categorized as containing small (still too faint to be clearly visible), intermediate or high proportion of WML. Those containing a small proportion of lesion are usually outside the “decision-threshold” of conventional segmentation, and point to early-stage WML.

The focus in the present study is to observe how the different stages of lesions are related to cognitive performance in a sample of elderly subjects with mild to moderate WML. The data used consisted of MRI measurements collected in a 3-year follow-up period, and annual neuropsychological assessments within that period. In particular, we were interested in determining whether even the earliest-stage small partial WML volumes, in normal-appearing brain tissue, are able to independently predict future cognitive decline, incremental to the conventionally evaluated WML load.

## Methods

### Subjects and design

The subjects were a subgroup of participants (*n* = 78) from three centers (Amsterdam *n* = 21, Graz *n* = 18, Helsinki *n* = 39) of the LADIS study, a European multicenter study investigating the impact of age-related WML in transition from functional independence into disability. The LADIS protocol and the sample characteristics have been reported in detail elsewhere (Pantoni et al., 2005). In short, 639 subjects were enrolled in 11 centers according to the following inclusion criteria: (a) age 65–84 years, (b) mild to severe WML according to the revised Fazekas scale (Pantoni et al., 2005), (c) no or minimal impairment in the Instrumental Activities of Daily Living scale (≤ 1 of 8 items compromised) (Lawton and Brody, 1969), and (d) presence of a regularly contactable informant. Exclusion criteria were: (a) severe illness likely prone to drop-out from follow-up (cardiac, hepatic, or renal failure, neoplastic, or other relevant systemic disease), (b) severe unrelated neurological illness or psychiatric disorder, (c) leukoencephalopathies of non-vascular origin (immunologic-demyelinating, metabolic, toxic, infectious), and (d) inability or refusal to undergo MRI scanning.

The baseline evaluation included brain MRI and thorough medical, functional, and neuropsychological assessments. The clinical assessments were repeated in 12-month intervals at three subsequent follow-up evaluations.

To allow for a valid comparison between subjects/centers, the MRI sequences obtained in each center had to be the same, and each patient had to have three sequences available, without major artifacts. The 78 subjects included in this study did not differ from the complete LADIS cohort in age, sex, baseline Mini-Mental State Examination (MMSE) score, or WML volume, but they had significantly higher education (9.3 vs. 11.7 years; *t* = −4.6, *p* < 0.001).

The study was approved by the Ethics Committees of each participating center in the LADIS study (LADIS Study Group, 2011). All subjects received and signed an informed written consent. The collaborators of the LADIS study are listed in the Appendix II.

### MRI acquisition and standard volume assessment

All axial MRI scans used were acquired with 1.5T equipment, following the same protocol at each center, including magnetization transfer images (TE = 10–14 ms, TR = 740–780 ms), T2-weighted fast spin echo images (TE = 100–120 ms, TR = 4000–6000 ms), and fluid attenuated inversion recovery (FLAIR) images (TE = 100–140 ms, TR = 6000–10000 ms, TI = 2000–2400 ms). All sequences had a voxel size of 1 × 1 × 5–7.5 mm^3^, FOV = 250 and an interslice gap of 0.5 mm.

The extent of hyperintensities on white matter regions including the infratentorial region was evaluated on axial FLAIR images with a semi-automated volumetric analysis (V_FLAIR_) using a Sparc 5 workstation (SUN) (van Straaten et al., 2006). Lesions were marked and borders were set on each slice using local thresholding (home-developed software Show_Images, version 3.6.1). No distinction was made between subcortical and periventricular hyperintensities. Areas of hyperintensity on T2-weighted images around infarctions and lacunes were disregarded. The number of lacunes was recorded in the white matter and in the deep gray matter using a combination of FLAIR, magnetization prepared rapid-acquisition gradient-echo, and T2 images to distinguish lacunes from perivascular spaces and microbleeds (Gouw et al., 2008). In addition, brain atrophy was rated according to a template-based rating scale on FLAIR images separately on cortical and subcortical regions (Jokinen et al., 2012).

### Image preprocessing

To guarantee that the multispectral information contained in each voxel originated from the exact same location in each subject, intra-patient registration was applied for all sequences available, using the SPM5 toolbox (Friston, 2003), and applying an affine transformation with the lowest resolution image, typically FLAIR, as the template. Furthermore, extra-meningeal tissue voxels were masked out, using a standard automatic method (BET2) (Smith et al., 2004).

### Discriminative clustering tissue segmentation

Recent advances in machine learning techniques have shown competitive results in tissue segmentation, often overcoming the accuracies achieved by classic region-growing or threshold-based methods (Styner et al., 2008). In particular, when compared to manual delineation, they are more robust and less subjective. The tissue segmentation method used in this study was such a machine learning technique, based on a data-driven self-supervised methodology, rooted on a DC strategy (Gonçalves et al., 2014). Similar to unsupervised clustering algorithms, such as k-nearest neighbors, DC groups input data according to their multi-dimensional gray level distributional information. In the current study, those distributions were three dimensional, which correspond to the total number of sequences used. The major asset of DC is its ability to use a small set of labeled information to support the clustering assignment. This feature leads to a clear improvement of the segmentation results, beyond traditional clustering techniques (Gonçalves et al., 2014).

The overall goal of DC can then be summarized as to partition the data space into clustered regions with rather uniform distributions throughout, and consistent label information for all voxels belonging to each cluster. A more detailed explanation is given in Appendix I, with the full mathematical description presented in Gonçalves et al. (2014).

### Partial volume estimation

DC gives the probability of membership of each voxel to all tissue classes, allowing for the estimation of partial volume information. Since we intended to focus our study on lesioned voxels, we only analyzed those where the proportion of lesion tissue present is relevant.

In this study, three different lesion categories were identified, leading to a corresponding amount of volume estimation: the volume of voxels that have a HIGH (V_DC100_), INTERMEDIATE (V_DC66_), or SMALL (V_DC33_) probability of being lesion. V_DC100_ and V_DC66_ are the volumes where the main tissue in the voxels therein, has a probability of being lesion of >66% and < 66%, respectively. Since both volumes V_DC100_ and V_*DC*66_ contain a majority of lesion tissue, V_FLAIR_^1^ can be approximated by the sum: V_FLAIR≈_V_DCHARD_^2^ ≥ V_DC100_ + V_DC66_. Hence, using DC, the best possible estimate of the volume of visible lesion is attained by V_DCHARD_. The last category, V_DC33_ corresponds to the volume of voxels where lesion is the second most probable tissue type, with probabilities ≥ 33%. Note that, this volume is not considered as lesion in normal segmentation methods, such as the one estimating V_FLAIR_, since lesion is never the main tissue type therein.

The ability of the present segmentation method to detect early-stage lesions was verified in a subgroup of patients (*n* = 19) with follow-up MRI data, *c.f*. Supplementary Materials: Appendix I). There we show that small partial WML volumes indicate possible future locations of fully developed lesions.

### Neuropsychological evaluation

The cognitive test battery of the LADIS study included the MMSE (Folstein et al., 1975), the Vascular Dementia Assessment Scale–Cognitive Subscale (VADAS) (Ferris, 2003), the Stroop test (MacLeod, 1991), and the Trail making test (Reitan, 1958). For the present purposes, we used the MMSE and VADAS total scores as global measures of cognitive function. In addition, three psychometrically robust compound measures were constituted for the evaluation of specific cognitive domains using averaged standard scores of the individual subtests as described previously (Moleiro et al., 2013): (1) *speed and motor control* = z scores (Trail making A + maze + digit cancellation)/3; (2) *executive functions* = z scores of [(Stroop III-II) + (Trail making B-A) + symbol digit modalities test + verbal fluency]/4; and (3) *memory* = z scores (immediate word recall + delayed recall + word recognition + digit span)/4.

The proportion of missing values in neuropsychological test variables varied between 0 and 6.4% at baseline, and between 24.4 and 32.1% at the last follow-up evaluation. This loss of data was due to subjects' death (*n* = 2), drop-out from the follow-up neuropsychological assessments (last year visit, *n* = 17), or inability to complete the entire test battery (*n* = 6).

### Statistical analysis

The predictors of longitudinal cognitive performance were analyzed using linear mixed models (restricted maximum likelihood estimation), which are able to deal with missing values and complex covariance structures. The assessment year (baseline, 1st, 2nd, and 3rd) was used as a within-subject variable, and unstructured covariance structure was adopted. The cognitive test scores were set as dependent variables. The partial lesion volumes (V_DC33_, V_DC66_, and V_DC100_) were tested as predictors, one by one. In all models, age, sex, and years of education were used as covariates. The models were repeated by adding V_FLAIR_ as another covariate, to find out the predictive value of the partial volume measurements incremental to that of the conventionally evaluated WML volume. Similarly, study center was added as a potential confounder, but since it had no essential effect on the results, it was left out from the final analyses. Because of skewed distributions possibly compromising the linearity assumption of the mixture models, logarithmic transformation was applied to all three partial volume measurements and V_FLAIR_. The results were analyzed with IBM SPSS Statistics 22 mixed module. Statistical significance was set at *p* < 0.05 for all the analyses.

## Results

### Characteristics of the subjects

The characteristics of the subjects at baseline are given in Table 1. According to the revised Fazekas scale, 28 (35.9%) subjects had mild, 26 (33.3%) moderate, and 24 (30.8%) severe WML.

**Table 1:** **Baseline characteristics of the subjects, *n* = 78**.

**Characteristic**	**Values**
Age, year, mean (SD)	74.2 (4.8)
Women, n (%)	45 (57.7)
Education, year, mean (SD)	11.7 (4.3)
MMSE score, mean (SD)	27.3 (2.5)
V_FLAIR_, mm^3^, mean (SD)	23.3 (22.3)
Dice score between V_FLAIR_ and V_DCHARD_, mean (SD)	70.3 (13.4)
**PARTIAL WML VOLUME MEASUREMENTS, CM^3^, MEAN (SD)**	
V_DC33_ (33%)	3.0 (2.6)
V_DC66_ (66%)	5.1 (5.1)
V_DC100_ (100%)	18.5 (20.6)

*MMSE, Mini-Mental State Examination; V_DC33_, volume of voxels containing small proportion of lesion; V_DC66_, volume of voxels containing intermediate proportion of lesion; V_DC100_, volume of voxels containing complete proportion of lesion; V_DCHARD_ = V_DC100_ + V_DC66_; V_FLAIR_, white matter lesion volume as measured with conventional semi-automated analysis on FLAIR images*.

### Partial WML volumes and other MRI findings

**Figure 1:**
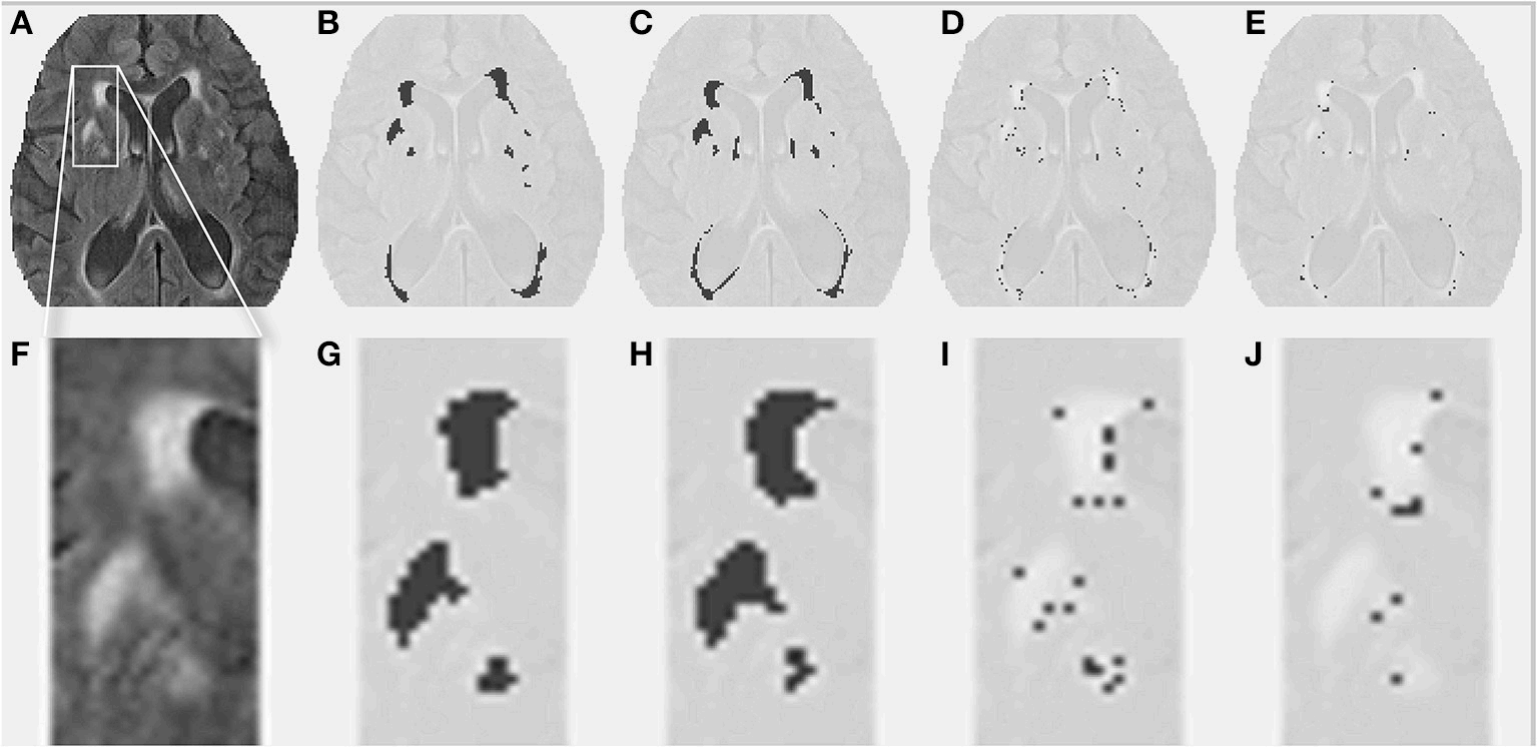
**White matter lesions (WML) at a middle level height. (A)** FLAIR image for a given subject. **(B)** Conventionally estimated WML. **(C–E)** Estimated WML, using the proposed segmentation algorithm, for full, intermediate, and small proportion of lesion. **(F–J)** Similar images for the zoomed portion depicted by the white box in **(A)**.

**Figure 2:**
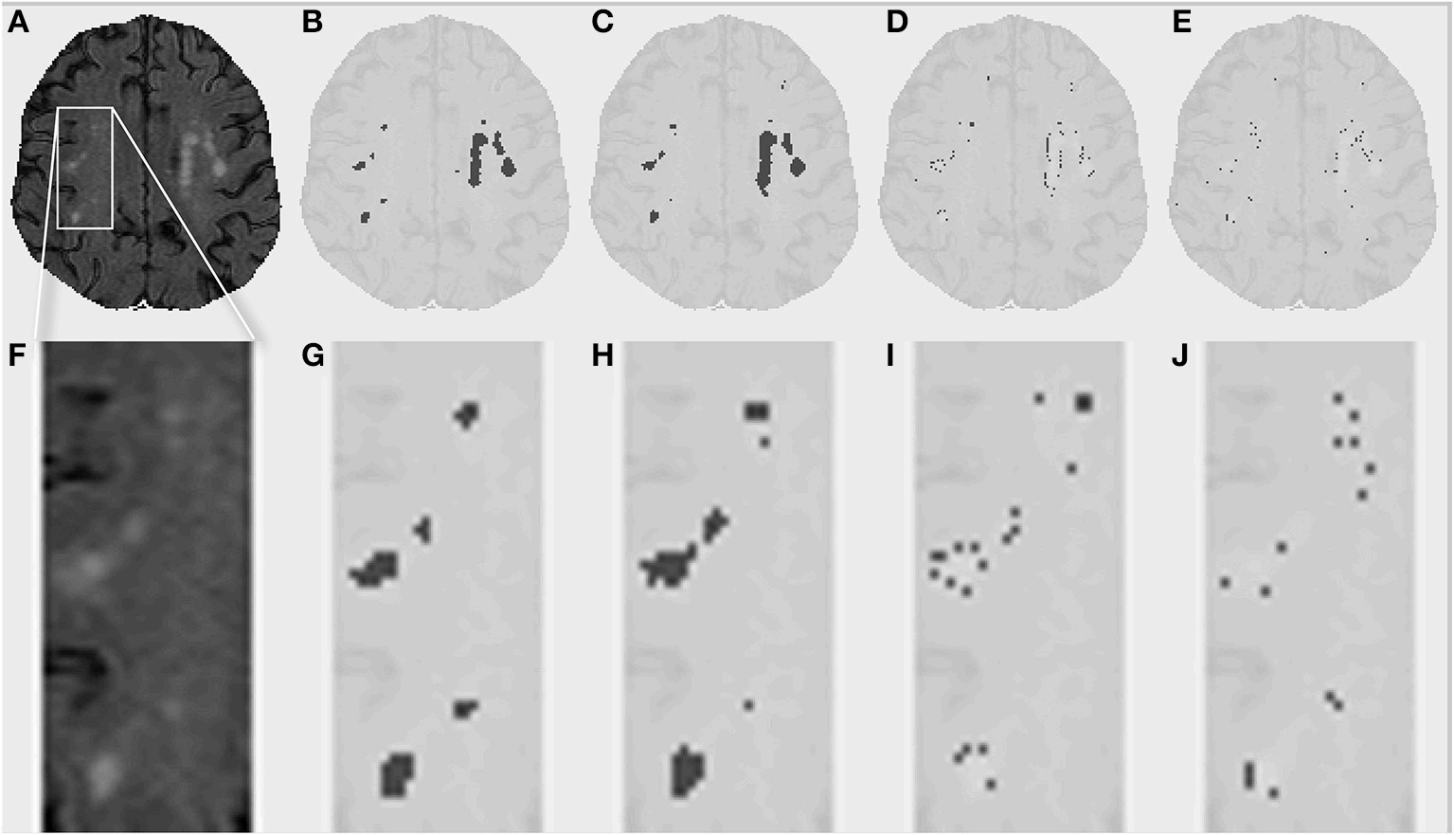
**White matter lesions (WML) in the centrum semiovale. (A)** FLAIR image for a given subject. **(B)** Conventionally estimated WML. **(C–E)** Estimated WML using the proposed segmentation algorithm, for full, intermediate, and small proportion of lesion. **(F–J)** Similar images for the zoomed portion depicted by the white box in **(A)**.

Table 1 shows the volumes obtained by the conventional segmentation method, the partial lesion volumes estimated by DC, and the Dice similarity coefficient comparing both segmentation methods. Figure 1 presents a comparison between the original FLAIR image (1A), the conventionally estimated hyperintensity volume, V_FLAIR_ (1B), and the results obtained for partial WML volumes V_DC100_ (1C), V_DC66_ (1D), and V_DC33_ (1E). Frames 1F-1J shows the corresponding images in the zoomed area denoted by the white rectangle of frame 1A. The evolution around the foci of lesion, from fully blown in the center to the intermediate stage and small proportion of lesion at the edges, can be seen in frames 1H–J. Note that the voxels classified as V_*DC*33_ are not included in V_FLAIR_, but are indicative of possible locations of future lesions. Figure 2 shows similar findings on a higher, centrum semiovale level. The DC segmentation procedure used three different sequences (FLAIR, T2, T1). Here, only FLAIR is shown for illustrative purposes.

For the whole dataset used, the three partial WML volume measures correlated significantly with each other: V_DC33_^*^V_DC66_ *r* = 0.87; V_DC33_^*^V_DC100_ *r* = 0.47; V_DC66_^*^V_DC100_ *r* = 0.47 (*p* < 0.001). They also correlated significantly with V_FLAIR_: V_DC33_ *r* = 0.26 (*p* = 0.024), V_DC66_ *r* = 0.26 (*p* = 0.023), and V_DC100_ *r* = 0.87 (*p* < 0.001), respectively. However, the measures were not significantly associated with presence of lacunar infarcts (no/few/many) or global brain atrophy score (cortical and subcortical) (*p* > 0.05).

**Figure 3:**
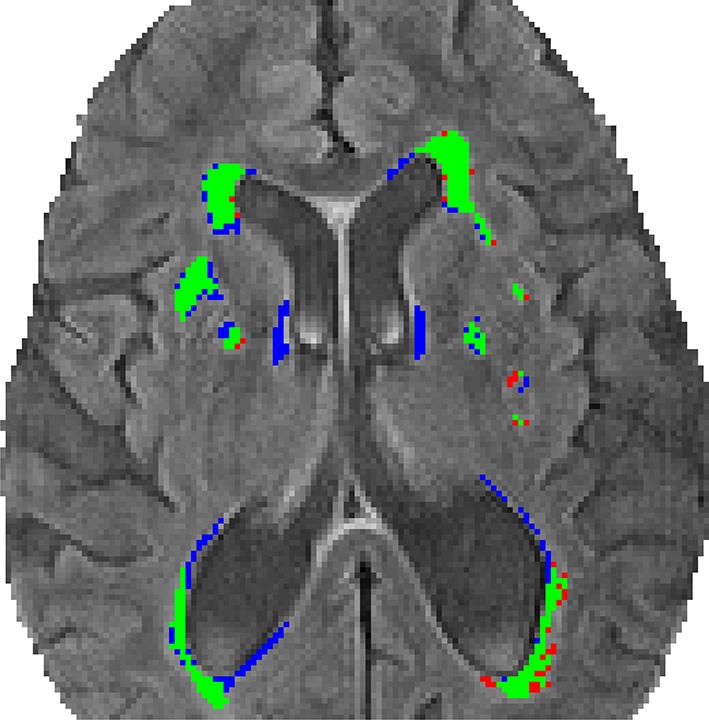
**Comparison of the segmentation methods**. This image shows the segmentation obtained using the semi-automated volumetric analysis (V_FLAIR_) and the discriminative clustering (V_DCHARD_) for the subject of Figure 1. The regions depicted in green correspond to the overlapping segmentation between both approaches. In red are shown regions classified as lesion only by the conventional method, while blue corresponds to voxel classified as lesion only by DC.

Figure 3 identifies the shared and disparate segmentations between the conventional segmentation (V_FLAIR_), and DC (V_DCHARD_) for the subject of Figure 1. There is a clear overlap between the two segmentations, as shown by the large number of green pixels. For the subject shown in that figure, there is a small difference between V_FLAIR_ and V_DCHARD_.

Estimating one subject's full tissue classification, using DC on a PC with Intel® Core™ i5-4590 CPU^@^ 3.30 GHz with 16 GB of RAM, took about 25 min. The estimation of the labels, on said computer, took about 70 min. An improvement of the latter estimation should streamline the procedure significantly.

### Partial WML volumes as predictors of cognitive performance

The relationships between partial WML volumes and longitudinal cognitive performance are summarized in Table 2. Linear mixed models adjusted for age, sex, and education showed significant negative associations between V_DC33_ and the compound score for executive functions. Firstly, V_DC33_ was associated with a significant main effect on overall level of executive performance (scores on average across all four temporal assessments). Secondly, the interaction between V_DC33_ and time (assessment year) indicated significant predictive value of V_DC33_ on change in executive performance over the 3-year follow-up. Specifically, higher load of V_DC33_ related to poorer performance at baseline and steeper decline in executive functions at each subsequent assessment year. After additional adjusting for V_FLAIR_, these results remained unchanged. Moreover, there was a weak baseline association between V_DC33_ and VADAS total score, but this result was no longer significant after controlling for V_FLAIR._V_DC33_ had no significant main effects or interactions with time in MMSE, VADAS, processing speed, or memory functions.

**Table 2:** **Relationship between partial white matter lesion volumes and cognitive performance in the 3 year follow-up**.

**Partial volumes**	**Cognitive performance**				
	**MMSE**	**VADAS**	**Speed**	**Executive**	**Memory**
**V_DC33_**					
Main effect	ns	ns	ns	12.1 (0.001)^**^	ns
Volume^*^time	ns	ns	ns	4.1 (0.011)^*^	ns
Per year	ns	bl	ns	bl^**^, 1 yr^**^, 2 yr^**^, 3 yr^**^	ns
**V_DC66_**					
Main effect	ns	4.9 (0.030)	ns	12.3 (0.001)^**^	ns
Volume^*^time	ns	ns^*^	3.2 (0.029)^*^	ns	ns
Per year	2 yr^*^	bl^*^, 1 yr^**^	ns	bl^**^, 1 yr^*^, 2 yr^*^, 3 yr^*^	ns
**V_DC100_**					
Main effect	13.1 (<0.001)^**^	20.6 (<0.001)^**^	7.0 (0.010)	28.9 (<0.001)^***^	4.8 (0.031)^*^
Volume^*^time	2.8 (0.047)^*^	ns	6.5 (0.001)^***^	5.7 (0.002)^**^	2.8 (0.050)
Per year	bl, 2 yr^**^, 3yr^*^	bl^***^	bl, 1 yr^*^	bl^***^, 1 yr^**^, 2 yr^***^, 3 yr^***^	bl^*^, 2 yr^*^, 3 yr^*^

*Linear mixed models adjusted for age, sex, and years of education*.
*Row, main effect F (p-value) indicates the association between partial lesion volume and mean cognitive performance across all time points*. Row, partial lesion volume^*^time interaction F (p-value) indicates the association with change of cognitive performance during follow-up. *Row, significant association at each time point; bl, baseline, 1 yr/2 yr/3 yr, change per each year compared to baseline (p < 0.05)*.

* *p < 0.5*,

** *p < 0.01*,

*** *p < 0.001; statistically significant after additional adjustment for V_FLAIR_*.

*MMSE, Mini-Mental State Examination; ns, non-significant; VADAS, Vascular Dementia Assessment Scale–Cognitive Subscale; V_DC33_, volume of voxels containing small proportion of lesion; V_DC66_, volume of voxels containing intermediate proportion of lesion; V_DC100_, volume of voxels containing complete proportion of lesion; V_FLAIR_, white matter lesion volume as measured with conventional semi-automated analysis on FLAIR images*.

V_DC66_ was related to significant main effects indicating poorer overall level of performance in VADAS and executive functions. Interaction between V_DC66_ and time was significant only for processing speed. Inspection of the results at individual time points showed a significant baseline association (VADAS, executive functions) as well as longitudinal change by the first (VADAS, executive functions), second (MMSE, executive functions), and the third (executive functions) follow-up year. Controlling for V_FLAIR_ had minimal effect on these results (Table 2).

Finally, V_DC100_ was associated with significant main effects in all neuropsychological scores. V_DC100_^*^ time interactions indicated a significant relationship with change during follow-up in four out of five cognitive measures. At this stage, the lesions were systematically associated with cognitive performance already at baseline. Moreover, steeper decline of performance was evident from the first to the last follow-up evaluation with some variation in different cognitive measures. Most of these results remained even after additional controlling for V_FLAIR_ despite its high correlation with V_DC100_ (Table 2).

Despite V_DC33_ and V_DC66_, V_FLAIR_ remained a significant predictor on overall performance over the follow-up period in VADAS and executive functions. However, V_FLAIR_ had no independent predictive value incremental to that of V_DC100_ on any of the cognitive measures.

## Discussion

This study examined the longitudinal cognitive impact of partial WML, from the faintest changes in normal-appearing white matter to the fully developed lesions. The investigation used a novel self-supervised multispectral MRI tissue segmentation method based on DC (Gonçalves et al., 2014) and annually repeated neuropsychological evaluations in 3-year follow-up. Different tissue types were identified utilizing all available MRI sequences simultaneously. WML was then categorized according to partial volumes as small, intermediate, and complete lesion.

Unlike conventional manual tissue segmentation, where the decision is based on an implicit gray level threshold, the proposed method gives access to “under-the-threshold” information regarding lesions. This allows for a better assessment of the lesion progression (qualitative information), as well as sub-voxel volumetry (quantitative information). Other methods exist that provide information about tissue proportions (Van Leemput et al., 2003; Manjón et al., 2010). Yet, they use certain priors that make them unsuitable for WML detection, such as the assumption that one voxel may not contain more than two tissue types.

The main finding of the present study was that even the smallest partial WML volume, V_*DC*33_, was significantly associated with poorer executive performance already at baseline and predicted future decline in executive functions over the 3-year follow-up. This effect was independent from demographic factors and, notably, also from the conventionally evaluated hyperintensity volume on FLAIR images. In a subgroup of subjects, we additionally showed that V_DC33_ likely represent the earliest changes in normal-appearing white matter, as their detection, at baseline, indicated future locations of the fully developed lesions after follow-up (Appendix I).

The intermediate stage lesions, V_DC66_, were independently associated with more extensive cognitive decline, including changes in processing speed and executive functions, as well as global cognitive functions. Moreover, the full-blown lesions, V_DC100_, were related to even more pronounced effects spreading to all evaluated cognitive domains, both at baseline and in follow-up. It is not surprising that V_DC100_ is a strong predictor of cognitive decline. Since V_DC100_ was highly correlated with V_FLAIR_, which has previously shown a strong association with cognitive change (Jokinen et al., 2011; Kooistra et al., 2014), it should hold rather similar predictive power.

The novel and most important outcome of the present research is that the volume of lesions detected below the decision threshold already allow for the prediction of particular cognitive scores. The earliest signs of cognitive decline were found specifically in executive functions, which are assumed to essentially rely on the integrity of the prefrontal-subcortical connections of the white matter (O'sullivan et al., 2001), Executive functions include cognitive control processes such as mental flexibility, inhibition, and planning related to complex goal-directed behavior. These functions are crucial to an individual's functional abilities in everyday life (Tomaszewski Farias et al., 2009).

The results presented in this article support the hypothesis that WML hyperintensities only represent “a tip of the iceberg,” while in fact white matter damage in SVD evolves as a gradual process affecting wider areas of the brain (Schmidt et al., 2011; Maillard et al., 2013). Diffusion imaging studies have shown that subtle microstructural changes, even in the normal-appearing brain tissue, are related to cognitive impairment and predict poor cognitive and clinical outcome in follow-up (Schmidt et al., 2010; Jokinen et al., 2013). Microstructural integrity is particularly reduced in the proximity of WML, as shown by fractional anisotropy (Maillard et al., 2011). This phenomenon called “WMH penumbra” may be related to the early-stage partial WML volumes observed in our study. Yet, early onsets of lesion may also occur at some distance from the fully developed WML, as illustrated in detail in Appendix I. To our knowledge, the relationship of these subliminal focal changes with cognitive outcome has not been shown before.

The present sample consists of a mixed group of older subjects, equally stratified to all WML severity degrees, from mild to severe. The participants were recruited in different settings, on the basis of varied referral reasons, representing the diversity of patients with WML encountered in clinical practice (LADIS Study Group, 2011). This heterogeneity of the subjects may, however, obscure the most subtle effects between imaging findings and cognitive decline. Typically to longitudinal studies on aging and cerebrovascular disease, some data was lost because of subjects' dropout from follow-up or inability to complete the entire evaluations.

As a limitation, the LADIS imaging protocol was not initially designed for the present quantitative segmentation method, so only a part of the original imaging data could be utilized. Furthermore, image noise, resolution and movement artifacts are all factors that may influence the outcome of a multicenter study like the one presented here. This is especially true when dealing with partial volume effects. In spite of these limitations, and after correcting for some of the aforementioned confounding factors, we were able to detect subtle indications of lesion progression, based on voxels with a small probability of being lesion.

To improve the reliability of the results shown in this manuscript, a larger cohort could have been considered. Due to concerns regarding consistency across centers, and changes in imaging setups at different times, a more strict policy should be used regarding the MRI sequences employed.

The strengths of this study include a novel, robust, self-supervised, and data-driven image analysis method that enables the identification of tissue types, and the quantification of pathological brain changes, at a very early stage, where conventional MRI evaluation would not be useful. The study also benefits from detailed neuropsychological evaluations, carried out at yearly intervals in 3-year follow-up.

In conclusion, early changes in the normal-appearing white matter already give a clue of progressive deterioration and poor cognitive outcome. At this stage, executive functions are primarily affected, but the detrimental effect on cognition becomes more global when the changes gradually develop into full-blown WML, eventually detectable also on conventional MRI tissue segmentation. These results affirm the proposed multispectral MRI tissue segmentation method as a promising tool having additive value in recognizing the risk of SVD and clinically significant progressive cognitive decline.

## Author contributions

All authors have made critical revisions of the manuscript for important intellectual content. In addition to that, the most central work of each author for the study was as follows: HJ; Responsible investigator and corresponding author, design and conceptualization of the study, neuropsychological and clinical data acquisition, statistical analysis, and interpretation, drafting and finishing of the manuscript. NG; Responsible investigator, design and conceptualization of the study, developing of the MRI segmentation method, MRI data analysis, drafting, and finishing of the manuscript. RV; Developing of the MRI segmentation method, MRI data analysis, design, and conceptualization of the study. JL; Expertise in statistical analysis and interpretation. FF; Design of the LADIS study, responsible for the MRI methods. RS; Design of the LADIS study, responsible for the MRI methods. FB; Design of the LADIS study, responsible for the MRI methods. SM; Construction of the neuropsychological test battery, neuropsychological and clinical data acquisition. AV; Neuropsychological and clinical data acquisition. DI; Study coordinator, member of the LADIS steering committee, design of the LADIS study. LP; Coordination and design of the LADIS study. TE; Member of the LADIS steering committee, design of the LADIS study, study conceptualization, and design. HJ and NG contributed equally to this work.

### Conflict of interest statement

The authors declare that the research was conducted in the absence of any commercial or financial relationships that could be construed as a potential conflict of interest.

## Footnotes

^1^V_FLAIR_, WML volume as measured with conventional semi-automated analysis on FLAIR images.

^2^V_DCHARD_, volume corresponding to the voxels where the majority of tissue is lesion.

## Abbreviations

DC: discriminative clustering
FLAIR: fluid-attenuated inversion recovery
LADIS: Leukoaraiosis and Disability Study
MMSE: Mini-Mental State Examination
MRI: magnetic resonance imaging
SVD: small vessel disease
VADAS: Vascular Dementia Assessment Scale–Cognitive Subscale
V_DC33_: volume of voxels containing small proportion of lesion
V_DC66_: volume of voxels containing intermediate proportion of lesion
V_DC100_: volume of voxels containing complete proportion of lesion
V_DCHARD_ = V_DC100_ + V_DC66_
V_FLAIR_: WML volume as measured with conventional semi-automated analysis on FLAIR images
WML: white matter lesion.
