# Supplementary material for: Early-Stage White Matter Lesions Detected by Multispectral MRI Segmentation Predict Progressive Cognitive Decline
Source: Front Neurosci. 2015 Dec 2;9:455. doi: 10.3389/fnins.2015.00455 (PMC4667087; doi:10.3389/fnins.2015.00455)
Supplement: Supplementary file 1 [file DataSheet1.DOCX]

## APPENDIX I

Progression of cerebral white matter lesions through self-supervised tissue segmentation

## I.1. Objective

A supplemental longitudinal MRI analysis was conducted, using the same data-driven self-supervised tissue segmentation methodology as in the main paper, rooted on a discriminative clustering (DC) strategy (Kaski et al., 2005). To identify various stages and the temporal evolution of white matter lesions (WML), this method estimates the probability of membership of each voxel to a set of expected brain tissue classes: white matter, grey matter and cerebrospinal fluid (CSF), as well as to WML. It works by maximizing the difference between tissue intensities, while minimizing the intensity variance within each class. By discretizing the probability of a voxel to belong to a particular tissue into small, intermediate or high probability, one can then assess how the various stages of disease progressed between recording instances.

There are several segmentation studies focusing on the time evolution of brain lesions. For example, in a 3 to 12 month multi-spectral MRI study (Elliott et al., 2013), new foci of multiple sclerosis could be identified, using random forest decision trees. Here, unlike the aforementioned approach, we explicitly investigated if voxels with a small probability of being lesion turn into full-blown lesions within a 3-year follow-up period, and how lesion probabilities evolve in the same time period. The underlying assumption to be tested is that weak probability of lesion reveals already the existence of structural changes, while indicating directions of evolution of the degenerative disease.

## I.2. Materials and Methods

### I.2.1. Subjects and design

The data in this appendix is a subsample of the one used in the main part of the manuscript. Due to the specific data requirements of this study, only a small subset of 19 patients, from one center (Helsinki), could be analyzed^[[1]](#footnote-1)^. First, data needs to be multi-spectral, i.e. more than one MRI sequence for each patient. Furthermore, all other acquisition properties should be identical at all measurement times. This means that the sequences analyzed at different time points should have an intensity distribution as similar as possible, and technical constraints, such as variations of head positioning, gradient non-uniformities and other sources of noise reduced to a minimum. The baseline characteristics of the subjects are presented in Table I.1.

Table I.1: Baseline characteristics of the participants (n=19)

| Characteristics | Values |
| --- | --- |
| Age, yr | 73.0±3.2 |
| Women | 14 (74 %) |
| Education, yr | 10.8±4.2 |
| White matter lesion score |  |
| Mild (punctate foci) | 5 (26%) |
| Moderate (beginning confluence of foci) | 8 (42%) |
| Severe (large confluent areas) | 6 (32%) |
| Data are represented as mean±SD or n (%). White matter lesions are depicted according to the modified Fazekas visual rating scale (Pantoni et al., 2005) | |

The baseline evaluation included brain MRI and thorough medical, functional and neuropsychological assessments. The latter assessments were repeated in 12-month intervals at 3 subsequent follow-up evaluations, while the time difference between the first MRI acquisition and the second one was of 3 years. Henceforth, we denote the time of the first and the second acquisitions as t_0_ and t_3_, respectively.

### I.2.2. MRI acquisition

The MRI scans were acquired with a 1.5T equipment, following the same protocol at both time acquisitions (van Straaten et al., 2006). These included axial T2-weighted fast spin echo images (TE=100-120 ms, TR=4000-6000 ms, FOV=250 mm, slice thickness: 5 mm, interslice gap 0.5 mm); FLAIR images (TE=100-140 ms, TR=6000-10000 ms, TI=2000-2400 ms, FOV=250 mm, slice thickness 5 mm, interslice gap 0.5 mm); and MT images (TE=10 ms, TR=760 ms, FOV=250 mm, slice thickness 5 mm, interslice gap 0.5 mm).

### I.2.3. General pre-processing

To perform voxel-by-voxel comparisons between images acquired at t_0_ and t_3_, the first step was to align all images with each other. All the sequences, at each acquisition time, were registered using the FLAIR sequence, at t_0_, as the reference. This procedure was done with the statistical parametric mapping toolbox (SPM5^[[2]](#footnote-2)^), using the default parameters. After registration, the next step was to mask out all the voxels outside the extra-cortical CSF. This was done using a standardized automatic method (BET2) (Hartley et al., 2006), also with its own default parameters.

### I.2.4. Normalizing images across time

One of the major problems in follow-up studies relates to the technical and morphological variations between the MR images acquired at different time points; such as from brain anatomical changes, image misalignments or non-uniform field gradients. Even when following the same protocol at both acquisition times, as with the data used in this study, some additional normalization procedures need to be conducted for a systematic and consistent analysis of the images.

The first consideration in our follow-up study was to ensure that, for each subject, the extra-cortical mask applied in all sequences is the same. Since all images were registered to one another, an overall mask was created using

${MASK}={MASK}_{t0}\wedge{MASK}_{t3}$ **,**

**where** $MASK$ **is constructed as the logical “and” between** ${MASK}_{t0}$ **and** ${MASK}_{t3}$**, which are the masks found for the t_0_ and t_3_ sequences, respectively. This binary overall mask is *false* for any voxel outside the extra-cortical CSF.**

**The next step dealt with differences in intensity between same sequence images. As an example, a FLAIR sequence collected at t_0_ might have an overall mean intensity higher than the one obtained** at t_3_, as shown in Figure I.1A. Such variations do not reflect biological differences, but rather technical ones, hence the need for correction. To compensate said differences, a histogram adjustment was conducted. For each sequence, mean values were calculated at t_0_ (μ_0_) and t_3_ (μ_3_). The intensity values of each sequence were then shifted by ±||μ_0_-μ_3_||/2, to reach an overall mean of (μ_0_+μ_3_)/2. This effect is depicted in Figure I.1B. With the mean intensities corrected, the next step was to adjust the histograms to the full 255 grey scale available. In addition, during this last normalization step, the histogram at t_3_ is made to be as close as possible to the one of t_0_. In this histogram normalization, the histograms of sequences at t_0_ and t_3_ were made to approximately match the histogram of the sequences at t_0_ with the full range of intensity (0-255). An example of the complete histogram normalization result can be seen in Figure I.1C. Note that such a transformation, akin to the generalized histogram equalization, does not take into account "where" changes occur in the images acquired at t_3_, but rather "how much". Therefore, it does not compensate any local changes, such as the ones coming from high magnetic field gradients, neither those corresponding to subtle variations coming from evolutions in WML.

**
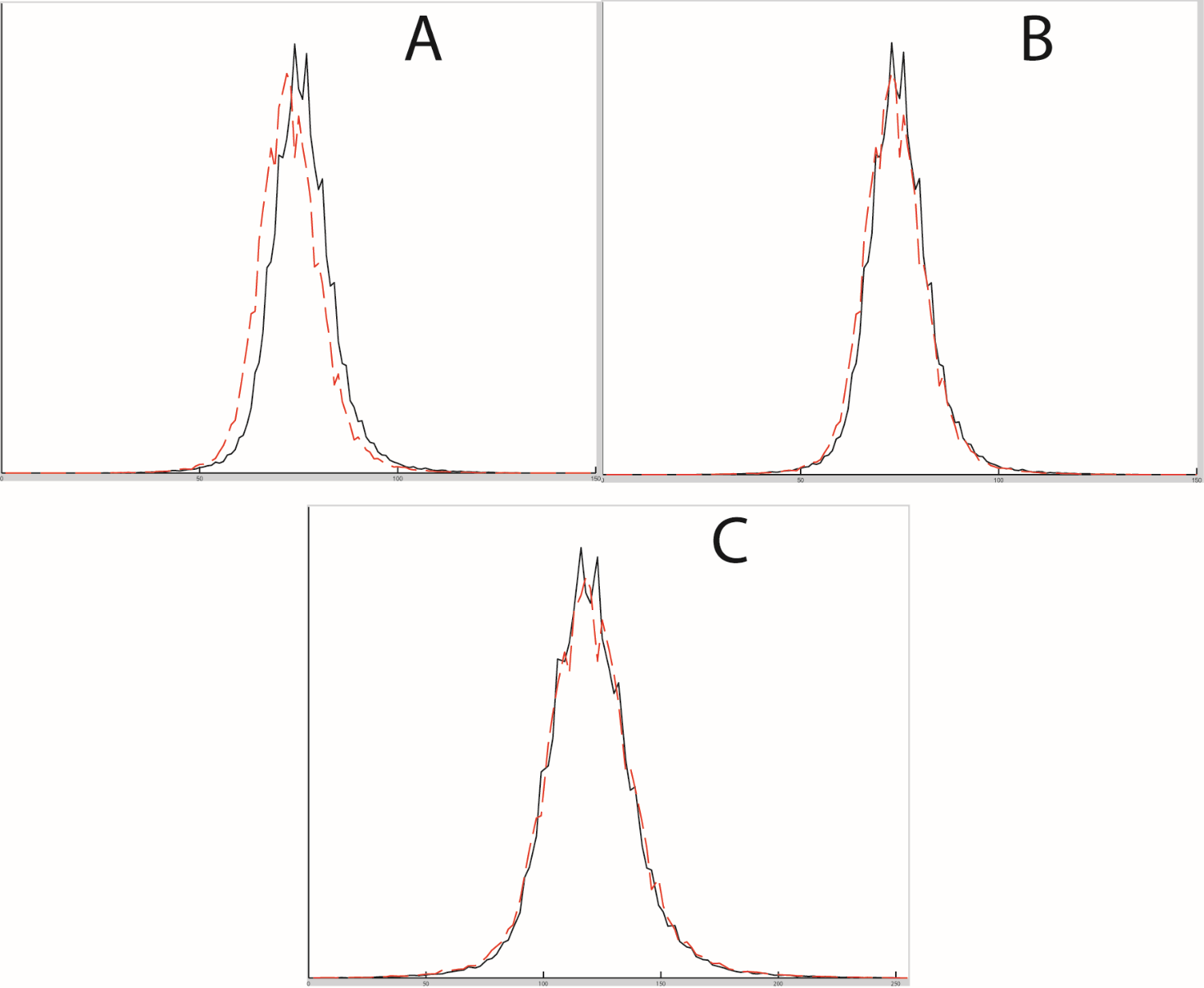
**

Figure I.1 Histogram adjustment. Frame (A) shows the original histogram of the FLAIR sequence for patient 1, while frame (B) depicts the mean-adjusted histogram. Frame (C) shows the normalized histogram.

The final step was to vectorise all images, and stack them, joining both t_0_ and t_3_ sequences in the same vector. This stacking was done to increase the number of total voxels of lesion available for segmentation, and to apply the exact same procedure to the data acquired at both times. The data took then the form of a matrix of observations, *D*, with 3 × *(Mt_0_+Mt_3_)*, where 3 is the number of modalities, and *M_ti_* corresponds to the number of voxels left after masking, for each recording. All rows of the observation matrix *D* were then normalized to lie between zero and one. This allowed us to study the relative variations of intensity values within a sequence, but not its global scale.



Figure I.2 shows the MR sequences for an exemplary subject, after all the pre-processing is done. The top row corresponds to t_0_, whereas the bottom one depicts t_3_. From left to right are FLAIR, T2 and MTI sequences. One can see that, besides the expected anatomical changes, there are other differences between images. The biggest difference is clearly visible when comparing both FLAIR sequences. The sequence taken at t_3_ exhibits a stronger effect of the non-uniformity field on the right-lateral side, when compared to the image collected at t_0_. The other sequences do not seem to suffer as much as FLAIR, having similar intensity behavior throughout.

Figure I.2 MR sequences for exemplary patient. Top row shows images collected at time t0 and bottom row at t3. From left to right, FLAIR, T2 and MTI sequences.

### I.2.5. Tissue segmentation

The tissue segmentation method used in this study was based on a data-driven self-supervised methodology, rooted on a DC strategy (Gonçalves et al., 2014). DC gives the probability of membership of each voxel to all tissue classes, allowing for the estimation of the proportion of lesion tissue present in a given voxel.

Similar to other clustering algorithms, the main goal of DC is to group input data according to their multi-dimensional distributional information. It works by partitioning directly the data space, using a combination of clustering and classification approaches. The first is similar to k-nearest neighbors, using Euclidean distance between image intensities, in which data samples closer to a cluster centroid contribute more to it than distant ones. To better estimate the tissue classes, DC also relies on a small amount of labels. The resulting regions should yield similar labeling outcomes for each cluster. Although the method requires only a small amount of labeled data, their existence is of paramount importance. Those voxel labels, corresponding to the different tissues to be segmented, are automatically estimated from the data itself, through an analysis of clustering consistency using self-organizing maps (Gonçalves and Vigário 2012).

One important distinction between DC and other clustering algorithms is that the correct number of DC clusters does not need to be pre-defined, and is not restricted to the number of tissue classes in the data. This allows for a better classification of tissue changes, since the “lesion” class can be assigned to more than one cluster, which may correspond to different stages in its development. Note also that this method is rather general, and not limited to the identification of a specific tissue type. In particular, it is capable of full-brain segmentation, hence not restricted to lesions found, e.g., inside white matter.

### I.2.6. Segmentation post-processing

Other anatomical changes may also occur, between t_0_ and t_3_, besides the WML. One such example are grey matter atrophies. These jeopardize the voxel-by-voxel comparison. To minimize such limitation, the Automatic Registration Toolbox (ART^[[3]](#footnote-3)^), was used to perform non-linear registration between the segmentation results obtained for t_0_ and t_3_ sequences. Using the FLAIR t_0_ as target and the FLAIR t_3_ as the image to be registered, a non-linear transformation was obtained. This transformation was then applied to the DC results for all sequences obtained at t_3_. We could then perform a voxel-by-voxel comparison between the results obtained, which resulted in a detailed analysis of the changes between categories, for each voxel, and their evolution between t_0_ and t_3_. We chose to only correct for these changes after the segmentation is performed, to avoid any local changes of intensities in the original images, which would bias the segmentation results. The segmentation was performed using the original intensities and not a modified version of them. Then we applied the transformation to the resulting segmentation.

## I.3. Results

### I.3.1. Volumetric results

A comparison between the volumes estimated for the three different lesion categories, at times t_0_ and t_3_, is presented in Table I.2, for all patients studied in this appendix (n=19).

Table I.2: Changes of the different partial lesion volumes

| Variation in the  number of lesion voxels (%) | 0___t_3_ | V_DC33__t_3_ | V_DC66__t_3_ | V_DC100__t_3_ | Sum |
| --- | --- | --- | --- | --- | --- |
| 0___t_0_ | --- | 78.9±8.6 | 65.5±13.1 | 32.4±18.3 |  |
| V_DC33__t_0_ | 50.5±16.1 | 6.8±5.7 | 13.6±8.6 | 29.1.0±13.9 | 100% |
| V_DC66__t_0_ | 47.6±18.1 | 5.4±3.1 | 14.5±9.9 | 32.5±12.6 | 100% |
| V_DC100__t_0_ | 25.9±20.3 | 2.2±1.4 | 8.8±7.4 | 63.1±19.9 | 100% |

0___t_0,_ volume not containing any lesion; V_DC33_, volume of voxels containing small proportion of lesion; V_DC66_, volume of voxels containing intermediate proportion of lesion; V_DC100_, volume of voxels containing high proportion of lesion

The label definitions in this table follow Sec. 2.5 of the main article

The entries in Table I.2 correspond to the percentage of changes in the volumes estimated at t_0_ and t_3_. The first row does not sum up to 100% since those values are actually the percentage of voxels that originated from non-lesion ones, at the other time point. From the other entries in the table, which are the focus of our analysis, we may see that, e.g., 6.8% of small probability voxels, at t_0_, remained so, whereas 13.62% progressed to intermediate and 30% to high lesion. The majority of lesion voxels with a small probability of being lesion at time t_0_ (V_DC33__t_0_) changed to either intermediate or high probability of lesion. As expected in this study, many new voxels were classified as lesion after the 3-year interval. Note that there are still some voxels that stopped being categorized as lesion from t_0_ to t_3_. Most of these cases are related to actual differences in the images, due to, for example, anatomical changes and differences in inhomogeneity behavior.

### I.3.2. Classification results


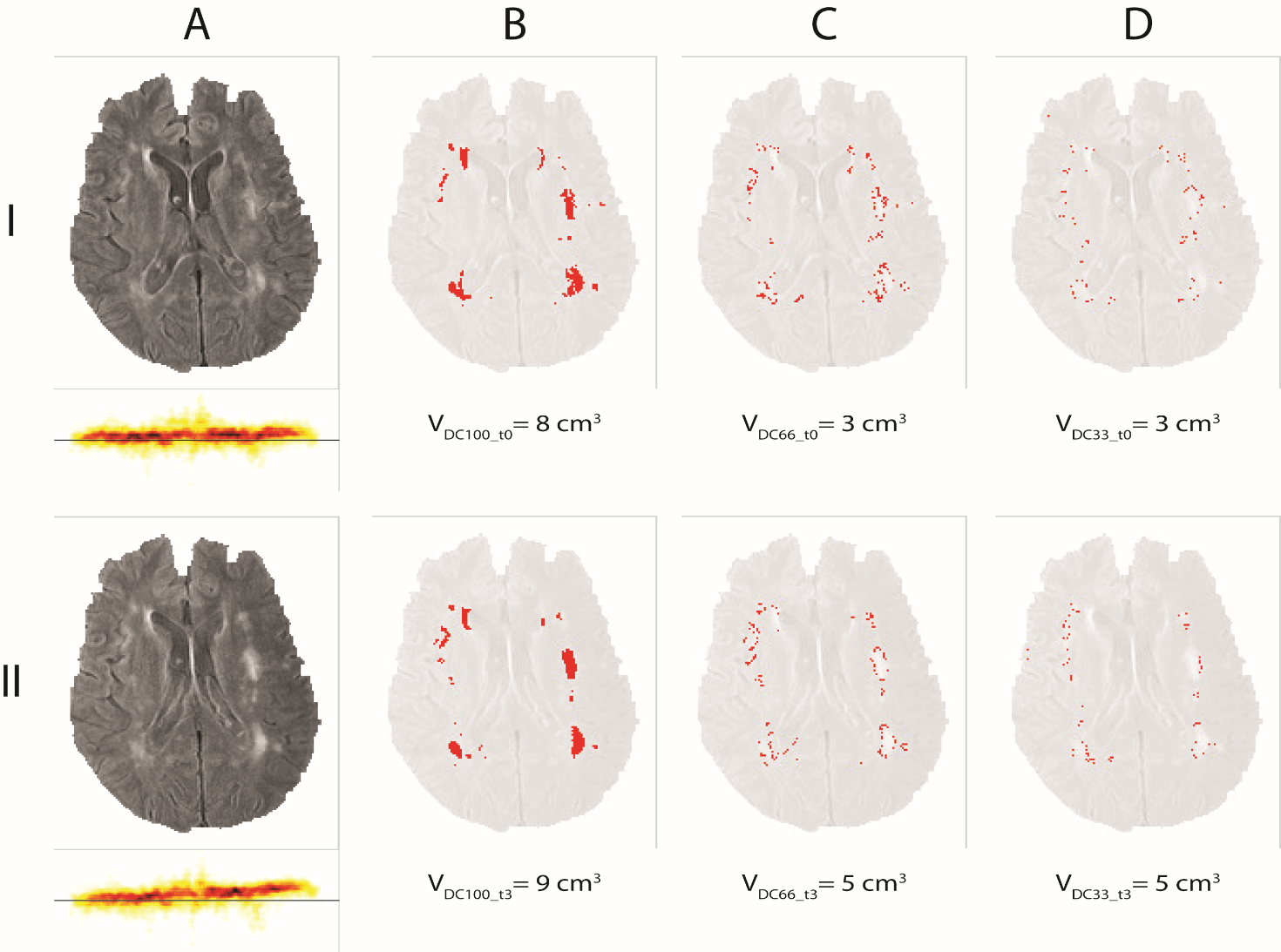
Figure I.3 shows the results obtained using the proposed method for the exemplary subject. The top row shows, from left to right, the FLAIR sequence, the voxels with high, intermediate and small probability of being lesion all at time t_0_, whereas the bottom row shows the same results for t_3_. Below the FLAIR sequences, a heatmap of intensity distribution is shown, while the lesion volume estimates are shown below the corresponding images. Most voxels with a small probability of being lesion at t_0_ have developed into full-blown lesion at t_3_. Furthermore, the lesion has spread according to the areas suggested at t_0_, by all three estimated volume areas.

Figure I.3 Results obtained for exemplary patient. Rows (I) and (II) show the images related to t_0_ and t_3_, respectively. Column (A) corresponds to FLAIR images, with the heatmap intensity distribution shown below it. Columns (B-D) show the estimated white matter lesions, for full, intermediate and small probability, respectively. Below the pictures is the total volume detected for each lesion probability.

## I.4 Discussion

This appendix focused on the evolution of WML by using pairs of MR images taken with a three-year interval. Particular focus was given to the ability to predict early-stage lesions that would develop into full-blown lesions, at the end of the follow-up period. Using a self-supervised multi-spectral MRI tissue segmentation method, based on discriminative clustering, and utilizing all available MRI sequences from both acquisition times, a volumetric and visual analysis of lesion evolution was conducted. This was possible due to the ability of DC to give tissue probabilities for each voxel, instead of a hard classification. By comparing the probabilities obtained for the images at the first time of acquisition with the ones from three years later, it was possible to study the evolution of lesions.

A similar study to ours (de Groot et al., 2013) focused on white matter change detection in the Rotterdam cohort study (Hofman et al., 2011). They found that white matter lesions develop gradually, and that quantifiable but not visible changes of the white matter precede the development of WML. This study used the Rotterdam Scan Study data set, comprising diffusion tensor imaging and FLAIR recordings. Unlike that study, we conducted our research on a cohort with a higher burden of WML. More importantly, the methodology proposed here takes into account partial volume information, allowing for the estimation of tissue probabilities.

Our results, which are in line with de Groot et al.'s, demonstrated that voxels with small probability of being lesion, in images taken at an early time point, tend to become full-blown lesions after three years. Maillard et al (Maillard et al., 2011) described the loss of micro-structural integrity in the proximity of WML as ``WMH penumbra''. We believe that the voxels with small or intermediate probability, near the main areas of lesion, can be related to such phenomenon. Nevertheless, we make use of a different concept for the definition of our "early stage" voxels than the aforementioned reference, since we consider tissue membership probability instead of partial volume. Therefore, early deteriorations of the white matter, as well as complete degeneration, but with a small volume proportion in the voxel will be considered, in our definition, as reflecting smaller membership probability. Furthermore, we also showed how the detection of lesion voxels and their probability assignments suggest possible future locations of lesions and how that lesion evolves with time. Such voxels tend to be sparsely distributed in the measured images and therefore of difficult detection. Nonetheless, they are of great importance when studying neurovascular disorders, since they open the possibility for early detection of pathological characteristics.

When dealing with age-related WML, a three-year timespan seems rather long, since lesions can go from a vestigial stage to fully blown in a significantly shorter time. Nonetheless, we showed that it is still possible to predict such changes, albeit with some limitations.

These limitations stem from several aspects; some related to the MRI technique itself, and others with the kind of follow-up analysis performed. The main problem in this kind of study is the differences between the sequences acquired at different time points. Even when using the same protocol, morphology changes in the brain, variations in the alignment of the brain with a reference, mechanical changes of the machine can cause discrepancies between acquisitions. All these problems will obviously influence the volumetric results and impair a proper voxel-by-voxel analysis. The nonlinear transformations used in this work were estimated using the complete brain information. Although we aimed at a rather global fit, this procedure cannot prevent the evolution of WML from contributing to the transformations' parameter estimation. It is expected that the limited extent of the lesions will mitigate the aforementioned influence.

Despite all the pre-processing corrections, one can still see voxels that were considered lesion at t_0_ but not at t_3_. This may be explained by comparing the FLAIR sequences in Fig. I.3 at both time stamps, and noticing the anatomical differences between them, that could not be fully compensated. For instance, there is a subtle difference displayed in the left posterior ventricular area, where one can see different anatomical structures. Similar problems can be caused by inhomogeneity field differences between acquisitions. An example of this can be seen in the clear slope shown on the FLAIR heatmap of Fig I.3 at t_3_, which is not visible at t_0_.

The method discussed here, which does not require an explicit anatomic or physiological model-prior, allowed for the identification of various stages of white matter lesions. By comparing processed images taken at different time instances, we showed how the detection of early stage changes in white matter suggests possible future locations of lesion, and how those lesions evolve with time.

Nonetheless, and in view of the limitations mentioned above, we recommend that further research is done with a more controlled data set, possibly utilizing earlier manifestations of WML, when the lesion has a somewhat more dynamical range of evolution, and with shorter intervals between follow-up measurements.

# I.5 references

de Groot, M., Verhaaren, B.F.J., de Boer, R., Klein, S., Hofman, A., van der Lugt, A.*, et al.* (2013). Changes in Normal-Appearing White Matter Precede Development of White Matter Lesions*. Stroke* 44, 1037-1042, doi: 10.1161/STROKEAHA.112.680223

Elliott, C., Arnold, D.L., Collins, D.L., and Arbel, T. (2013). Temporally Consistent Probabilistic Detection of New Multiple Sclerosis Lesions in Brain MRI*. IEEE Trans Med Imaging* 32, 1490-1503, doi: 10.1109/TMI.2013.2258403

Gonçalves, N., Nikkilä, J., and Vigário, R. (2014). Self-supervised MRI tissue segmentation by Discriminative Clustering*. Int. J. Neural Syst.* 24, 1450004, doi: 10.1142/S012906571450004X

Gonçalves, N. and Vigário, R. (2012). "Clustering through SOM Consistency," in *Proceedings of the 9th International Conference on Image Analysis and Recognition - Volume Part I*, eds. A. Campilho and M. Kamel (Aveiro, Portugal: Springer Berlin Heidelberg), 61-68

Hartley, S.W., Scher, A.I., Korf, E.S.C., White, L.R., and Launer, L.J. (2006). Analysis and validation of automated skull stripping tools: A validation study based on 296 MR images from the Honolulu Asia aging study*. Neuroimage* 30, 1179, doi: DOI: 10.1016/j.neuroimage.2005.10.043

Hofman, A., van Duijn, C.M., Franco, O.H., Ikram, M.A., Janssen, H.L., Klaver, C.C.*, et al.* (2011). The Rotterdam Study: 2012 objectives and design update*. Eur.  J.  Epidemiol.* 26, 657-686, doi: 10.1007/s10654-011-9610-5

Kaski, S., Sinkkonen, J., and Klami, A. (2005). Discriminative Clustering*. Neurocomputing* 69, 18-41, doi: http://dx.doi.org/10.1016/j.neucom.2005.02.012

Maillard, P., Fletcher, E., Harvey, D., Carmichael, O., Reed, B., Mungas, D.*, et al.* (2011). White matter hyperintensity penumbra*. Stroke* 42, 1917-1922, doi: 10.1161/STROKEAHA.110.609768

Pantoni, L., Basile, A.M., Pracucci, G., Asplund, K., Bogousslavsky, J., Chabriat, H.*, et al.* (2005). Impact of age-related cerebral white matter changes on the transition to disability -- the LADIS study: rationale, design and methodology*. Neuroepidemiology* 24, 51-62, doi: 10.1159/000081050

van Straaten, E.C., Fazekas, F., Rostrup, E., Scheltens, P., Schmidt, R., Pantoni, L.*, et al.* (2006). Impact of white matter hyperintensities scoring method on correlations with clinical data: the LADIS study*. Stroke* 37, 836-840, doi: 10.1161/01.STR.0000202585.26325.74

## APPENDIX II

The Leukoaraiosis and Disability (LADIS) Study: List of participating centers and personnel

Helsinki, Finland (Department of Neurology, Helsinki University Central Hospital and Department of Neurological Sciences, University of Helsinki, Finland): Timo Erkinjuntti, MD, PhD, Tarja Pohjasvaara, MD, PhD, Pia Pihanen, MD, Raija Ylikoski, PhD, Hanna Jokinen, PhD, Meija-Marjut Somerkoski, MPsych, Riitta Mäntylä, MD, PhD, Oili Salonen, MD, PhD; Graz, Austria (Department of Neurology and Department of Radiology, Division of Neuroradiology, Medical University Graz): Franz Fazekas, MD, Reinhold Schmidt, MD, Stefan Ropele, PhD, Brigitte Rous, MD, Katja Petrovic, MagPsychol, Ulrike Garmehi, Alexandra Seewann, MD; Lisboa, Portugal (Serviço de Neurologia, Centro de Estudos Egas Moniz, Hospital de Santa Maria): José M. Ferro, MD, PhD, Ana Verdelho, MD, Sofia Madureira, PsyD, Carla Moleiro, PhD; Amsterdam, The Netherlands (Department of Radiology and Neurology, VU Medical Center): Philip Scheltens, MD, PhD, Ilse van Straaten, MD, Frederik Barkhof, MD, PhD, Alida Gouw, MD, Wiesje van der Flier, PhD; Goteborg, Sweden (Institute of Clinical Neuroscience, Goteborg University): Anders Wallin, MD, PhD, Michael Jonsson, MD, Karin Lind, MD, Arto Nordlund, PsyD, Sindre Rolstad, PsyD, Ingela Isblad, RN; Huddinge, Sweden (Karolinska Institutet, Department of Neurobiology, Care Sciences and Society; Karolinska University Hospital Huddinge): Lars-Olof Wahlund, MD, PhD, Milita Crisby, MD, PhD, Anna Pettersson, RPT, PhD, Kaarina Amberla, PsyD; Paris, France (Department of Neurology, Hopital Lariboisiere): Hugues Chabriat, MD, PhD, Karen Hernandez, psychologist, Annie Kurtz, psychologist, Dominique Hervé, MD, Sarah Benisty, MD, Jean Pierre Guichard, MD; Mannheim, Germany (Department of Neurology, University of Heidelberg, Klinikum Mannheim): Michael Hennerici, MD, Christian Blahak, MD, Hansjorg Baezner, MD, Martin Wiarda, PsyD, Susanne Seip, RN; Copenhagen, Denmark (Memory Disorders Research Group, Department of Neurology, Rigshospitalet, and the Danish Research Center for Magnetic Resonance, Hvidovre Hospital, Copenhagen University Hospitals): Gunhild Waldemar, MD, DMSc, Egill Rostrup, MD, MSc; Charlotte Ryberg, MSc, Tim Dyrby MSc, Olaf B. Paulson, MD, DMSc; Ellen Garde, MD, PhD; Kristian Steen Frederiksen, MD; Newcastle-upon-Tyne, UK (Institute for Ageing and Health, Newcastle University): John O'Brien, DM, Sanjeet Pakrasi, MRCPsych, Mani Krishnan MRCPsych, Andrew Teodorczuk, MRCPsych, Michael Firbank, PhD, Philip English, DCR, Thais Minett, MD, PhD.

The Coordinating center is in Florence, Italy (Department of Neurological and Psychiatric Sciences, University of Florence): Domenico Inzitari, MD (Study Coordinator); Luciano Bartolini, PhD, Anna Maria Basile, MD, PhD, Eliana Magnani, MD, Monica Martini, MD, Mario Mascalchi, MD, PhD, Marco Moretti, MD, Leonardo Pantoni, MD, PhD, Anna Poggesi, MD, PhD, Giovanni Pracucci, MD, Emilia Salvadori, PhD, Michela Simoni, MD.

The LADIS Steering Committee is formed by Domenico Inzitari, MD (study coordinator), Timo Erkinjuntti, MD, PhD, Philip Scheltens, MD, PhD, Marieke Visser, MD, PhD, and Peter Langhorne, MD, BSC, PhD, FRCP who replaced in this role Kjell Asplund, MD, PhD beginning in 2005.

1. Note that the original study, from which we extracted the data used in this investigation, albeit very well controlled over centers and recording instances, did not have as stringent consistency requirements as the ones in the present study. [↑](#footnote-ref-1)
2. Fil Methods Group, 2005. Statistical parametric mapping - <http://www.fil.ion.ucl.ac.uk/spm>. [↑](#footnote-ref-2)
3. NITRC group, 2011. Automatic Registration Toolbox - <http://www.nitrc.org/projects/art/>. [↑](#footnote-ref-3)
